# Dielectrophoresis: An Approach to Increase Sensitivity, Reduce Response Time and to Suppress Nonspecific Binding in Biosensors?

**DOI:** 10.3390/bios12100784

**Published:** 2022-09-23

**Authors:** Anders Henriksson, Peter Neubauer, Mario Birkholz

**Affiliations:** 1 Chair of Bioprocess Engineering, Department of Biotechnology, Technische Universität Berlin, Ackerstraße 76, 13355 Berlin, Germany; 2 IHP—Leibniz-Institut für Innovative Mikroelektronik, Im Technologiepark 25, 15236 Frankfurt (Oder), Germany

**Keywords:** dielectrophoresis, biosensor, diffusion, mass-transfer, non-specific interaction, AC electrokinetics

## Abstract

The performance of receptor-based biosensors is often limited by either diffusion of the analyte causing unreasonable long assay times or a lack of specificity limiting the sensitivity due to the noise of nonspecific binding. Alternating current (AC) electrokinetics and its effect on biosensing is an increasing field of research dedicated to address this issue and can improve mass transfer of the analyte by electrothermal effects, electroosmosis, or dielectrophoresis (DEP). Accordingly, several works have shown improved sensitivity and lowered assay times by order of magnitude thanks to the improved mass transfer with these techniques. To realize high sensitivity in real samples with realistic sample matrix avoiding nonspecific binding is critical and the improved mass transfer should ideally be specific to the target analyte. In this paper we cover recent approaches to combine biosensors with DEP, which is the AC kinetic approach with the highest selectivity. We conclude that while associated with many challenges, for several applications the approach could be beneficial, especially if more work is dedicated to minimizing nonspecific bindings, for which DEP offers interesting perspectives.

## 1. Introduction

Since the report of the Clark electrode in the 1970s, breakthrough developments in biomolecule immobilization, signal transduction, and device integration have been achieved that have improved the performance as well as expanded the applications of biosensors [1]. The detection principle of the sensors generally requires that analytes in a solution interact with receptor molecules. These are often immobilized onto a sensor surface, although other sensor principles that, for example, rely on a volume-related detection have also been developed [2,3]. In this review, we focus on surface-based biosensors, however. The reaction is subsequently transduced into a measurable electronic signal whose amplitude correlates to the analyte concentration. The performance of the biosensor may be evaluated based on specificity, its limit of detection (LOD), and sensitivity, defined as the ability to measure small concentration changes of the analyte.

An approach to improve the transduction method of biosensors has been to utilize micro- and nano-scaled materials [4] such as nanomechanical systems [5], nanopore sensors [6,7,8], Nanowire field effect transistors NWFET’s [9], or surface-enhanced RAMAN [10]. Due to the miniaturization of the materials, surface properties and surface reactions gains importance allowing only few molecules to influence the inherent properties of the device such as refractive index [11,12], wettability [13], photoluminescence [14], or conductivity [15]. This enables highly sensitive and efficient transducers. The increased sensitivity of nanobiosensors has allowed even the detection of single molecules as well as stochastic behavior on the sensor surface [8,16]. Furthermore, the realization of these extremely sensitive sensors allows detection of analytes in concentration in the femtomolar and even attomolar range enabling early-stage disease diagnosis and individual adapted medical treatments.

As analyte concentrations become increasingly low, however, it becomes increasingly difficult to transport the few molecules in the solution to the sensor surface, often being a main factor determining the LOD that can be achieved on reasonable short timescales. 

Diffusion or Brownian motion is the most important matter of mass transfer by which biomolecular analytes eventually interact with the sensing surface. Brownian motion is the random uncontrolled movement of particles as a result of continuous collision with molecules of the surrounding medium. This may cause a net movement known as diffusion. The solutes move down a concentration gradient from an area of higher concentration to an area of lower concentration, with a time constant that correlates with the square of the distance that the diffusing species must travel. Furthermore, as the analyte interacts with the receptor on the sensor surface, the concentration of analytes near the surface is depleted, forming a depletion region. Without convection, the depletion region will grow over time as more analytes bind to the surface, following longer distances for the diffusion process [17]. The size and shape of the biomolecular analytes of interest, combined with physiological temperatures, dictate that in the minutes-to-hours timescale appropriate for rapid biomolecular detection, typical large biological analyte molecules can diffuse 10–100 μm [18]. Diffusion may be further hampered by steric hindrance, especially on nanostructured sensors and porous substrates [19]. 

The Brownian motion, characterized by the diffusion constant, is inversely proportional to the diameter of the particle. Accordingly, random displacement tends to be stronger on smaller particles below 100 nm while the Brownian motions of particles above 1 µm are often much smaller. In this way, it is even more difficult to analyze low concentrations of larger particles such as whole cells due to mass transfer limitation. The analysis of cells may be further hampered if the cells are actively moving.

Additionally, the affinity of the analyte to the receptor has an important influence on the sensing performance and the dissociation constant K_D_ of the analyte receptor complex is an important parameter. A high affinity of the receptor-analyte complex that exceeds nonspecific bonding of interfering molecules is a prerequisite for sensitive sensors with high specificity. While modern high performance sensors with low levels of background enable the quantification of molecules at concentrations far below of their K_D_ value, it is commonly estimated that conventional immunoassays still should only be implicated to quantify target concentrations within the range K_D_/9 and 9 × K_D_ [20]. Accordingly, streptavidin–biotin which is one of the stronger complexes with K_D_ values in the order of 10^−15^ M [21] would have a predicted detection limit in the femtomolar range whereas the detection limit of majority of antibody–antigen complexes would be in the nano to picomolar range [22]. To push LOD to lower levels with response times within minutes to a few hours, solutions for the reduction of background noise from nonspecifically bonded species as well as for accelerated diffusion are required.

The complex interplay between transport phenomena and reaction kinetics was modeled by Squires et al. [17]. For instance, they calculated a single binding event only every 3 days in a sensor, modeled as a nanowire with a diameter of 10 nm and length of 2 μm in a microchannel with a length and height of 100 um through which a target protein solution with a concentration of 10 fM flows. This example of a kinetic limited sensor highlights the importance of designing biosensor platforms with efficient mass transfer solutions. Without methods to actively direct biomolecules to a sensor surface, individual nanoscale sensors will at the best be subject to picomolar order detection limits, as concluded by Sheenan et al. [23].

One effective way to reduce the diffusion path and the response time of the sensor is to extend the sensor so that the sensing interface reaches further into the sample solution using nanostructured electrodes or magnetic nanoparticles [8].

Magnetic nanoparticles of various materials such as Fe_3_O_4_, MnFe_2_O_4_, CoFe_2_O_4_, CoPt_3_ with immobilized capture molecules may be dispersed in the sample solution. By using a magnet to collect the nanoparticles for measurements, the detection limit may be dramatically lowered as the majority of the analytes in the sample is collected by the nanoparticles [24].

Increasing the flux of analyte to a sensing interface via convection is another common method that has shown to reduce the diffusion layer and to be an efficient way to improve sensing performance. A large variety of such methods has been developed during the last decades, generally based on microfluidic systems with passive or active mixing [25]. Passive systems realize the mixing by virtue of their geometry and any natural flow features that arise. Active systems are defined as methods that force the fluid to behave in a manner that cannot be achieved through geometry alone. Therefore, the use of pumps and electric fields for reasons of mixing rather than simple locomotion would be classified as an active mixing system [25]. AC electrokinetic and its implications in biosensors is a growing field of research with proof of principle experiments that has shown a decrease of the limit of detection by several orders of magnitude and decreased the detection time from hours to minutes [26,27]. The most frequently applied methods to increase the mass transfer and achieve an enrichment of analytes on the sensor surface are AC electroosmosis, AC electrothermal effect [28,29] and dielectrophoresis (DEP). This review focuses on DEP. The advantage of this method in compare to the other AC kinetic approaches or other methods that can be applied to manipulate cells and particles in microfluidic systems such as magnetophoresis [30], acoustophoresis [31], and optical methods [32] is its high selectivity and controllability. A review comparing the different methods was recently puplished by Afsaneh et al. [33]. 

While previous reviews have covered generally AC electrokinetic enhanced biosensors [26,27], DEP in microbial sensors [34] or the detection of biomarkers [35], we are here aiming to cover biosensors assisted with DEP in the light of new theoretical approaches, recent examples and critically discuss their potential to increase sensitivity and to decrease the assay time of biosensors. Key aspects in this endeavor are an improved mass transfer and the avoidance of nonspecific bindings.

## 2. Principle of Dielectrophoresis

DEP is the movement of particles exposed to an inhomogeneous oscillating electric field, due the induced polarizability gradient between the particles and the suspending medium due to their intrinsic dielectric properties. The induced DEP-force of a spherical particle with diameter d may be described as:

(1) $$F_{DEP}=\frac{\pi }{4}d_{p}^{3}\;\cdot \;\varepsilon_{m}^{’}Re\left\{\frac{\varepsilon_{p}^{*}-\varepsilon_{m}^{*}}{\varepsilon_{p}^{*}+2\varepsilon_{m}^{*}}\right\}\;\cdot \;\nabla {\left|\vec{E}\right|}^{2}$$

In brackets is given the Clausius–Mosotti (CM) factor describing the dielectric properties of the particles (*p*) and the medium (*m*), expressed by a function of their complex dielectric constants $\varepsilon_{p}^{*}(\varpi )$ and $\varepsilon_{m}^{*}(\varpi )$. These functions depend on the real part of permittivity *ε*′ and electrical conductivity σ, and are therefore dependent on the frequency *f* or the angular frequency ω = 2π*f*, respectively, by which the applied voltage is oscillating, leading to *ε*^∗^ = *ε*′ + iσ/ω. The real part of the CM can be switched between positive and negative values (−0.5 to 1) by changing the frequency, resulting in negative DEP that pushes particles away from the highest E field or positive DEP (pDEP) that induces a particle movement towards the highest fields. The frequency dependency of F_DEP_ may be attributed to σ being the dominant factor describing CM at lower frequencies whereas *ε*′ is dominant at higher frequencies.

The magnitude of the local square of the electric field gradient (∇E^2^) is the second parameter that influences the interaction with a particle. The generated magnitude of ∇E^2^ depends on the applied voltage *V* as well as on the electrode geometry. The layout of the flow channel and the electrodes thus offer various degrees of freedom for choosing electrode configurations and electrode distances to generate strongest possible ∇E^2^. This is crucial especially for the interaction with smaller particles such as protein molecules. In addition to ∇E^2^, the magnitude of the other electrokinetic or hydrodynamic forces present in the system should be taken into consideration when completing the electrode design. In case the particle is exclusively exposed to DEP, we may expect that the required ∇E^2^ to observe a DEP interaction would be less intense compared to particles that simultaneously are exposed to electrophoresis, electroosmotic force, and pressure gradients. This is important, as electroosmosis and electrophoretic effects may be strongly present for many applications. When applying an inhomogeneous DC field, both forces make a significant contribution, but especially electroosmosis also contributes when applying an AC field below 1 MHz [36,37]. The frequencies generating the maximal electroosmotic fluid flow seems to be dependent on the on the electrode geometry in which the greatest fluid velocity for coplanar electrode geometries often appear at frequencies between 100 kHz to 1 MHz and in case the electrodes are placed orthogonal to each other, the maximum could be observed at lower frequencies [38]. Consequently, the electrode design is important, both to maximize the ∇E^2^ and controlling other electrokinetic forces. 

Based on the electrode set-up, DEP can be categorized as electrode-based DEP (eDEP) or insulator-based DEP (iDEP). Electrode based DEP utilize a pair of electrodes differing in size or shape upon which an alternating current (AC) voltage is applied to generate a nonuniform electric field. It is frequently used for manipulating particles in microfluidic devices as it generates high field gradients with low applied voltages. In an iDEP set-up, insulating structures such as posts, membranes, obstacles, or constrictions are built within the microfluidic channel, which deforms the applied electric field creating a high electric field gradient with a local maximum within the channel. The approach has been applied to trap a large variety of particles both by applying DC voltages and AC voltages. The advantage with this set-up is less generated joule heating and avoidance of electrochemical side effects [39]. It has been widely accepted that the DEP force is responsible for trapping the particles regardless of if an DC voltage or an AC voltage is applied. Recent work suggests, however, that electrophoresis and electroosmosis are the most important forces present when working in DC mode, and it is better referred to as DC insulator-based electrokinetic [40,41,42,43]. Readers should have this in mind when works on DC iDEP are cited in this review.

A correlation of the DEP force with the cubic of particles diameter makes particle volume the most important parameter for a strong DEP manipulation. Consequently, DEP has been a suitable method for size dependent discrimination of a large variety of particles [44].

**Figure 1:**
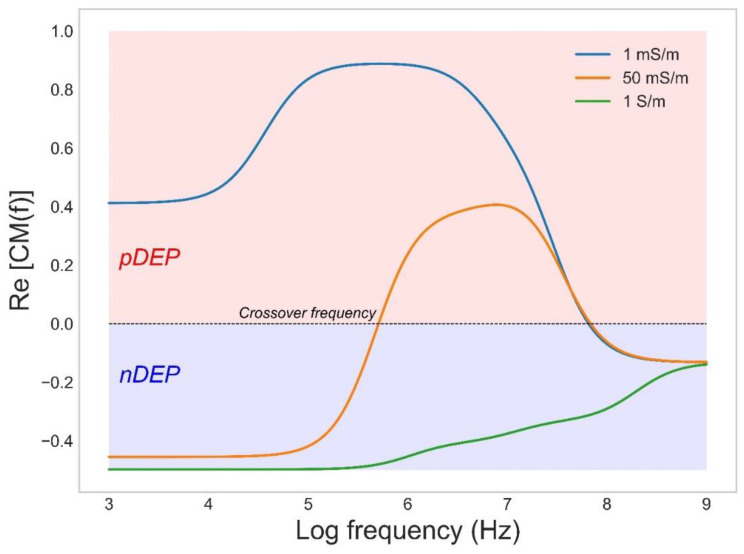
The CM factor as a function applied frequency for the bacterium *Escherichia coli* in medium with conductivities of 1, 50, and 1000 mS/m.

In biotechnology DEP has been frequently applied for separation of various microscopic scaled particles such as blood cells [45], microalgae [46,47], yeast [48], and bacteria [34]. This allows applications such as cell separation and sorting, concentration, and cell trapping [34]. For predicting the DEP force of a cell, the cell may be considered as a spherical particle with single or multiple shells. The simplest model includes a low conductive cell wall around a conducting cytoplasmic region where the dielectric properties of each part (cytoplasm, membrane, and wall) can be described by its conductivity and permittivity. For most cells suspended in a low conducting medium (below 1 mS/m), the field causes pDEP at frequencies below 10 MHz whereas the field in high conducting medium (over 100 mS/m) causes nDEP over all frequencies. Moderate conductive medium causes a more dynamic DEP response (Figure 1). At low frequencies, the field is mainly blocked by the cell wall and membrane, which causes an nDEP behavior, while the field permeates deeper at higher frequencies and may cause cytoplasmic polarization; hence, gradually a shift to pDEP may be observed. At even higher frequencies, the insufficient time available for cytoplasmic polarization causes the pDEP level to gradually fall again, whereas the contribution of permittivity terms starts to dominate, and eventually a switch back to nDEP can be observed. 

Apart from the radius of the particles, cells can also be separated based on different parameters that affect its dielectric properties, such as lipid content in microalgae [49], or its surface properties [50]. For example, recent work by Buie et al. has shown a strong correlation between the ability of electrotroph bacteria to accept surface electrons via so-called extracellular electron transfer (EET) and its surface polarizability. This work holds exciting promise for rapid screening of direct EET via a noninvasive dielectrophoretic screening process [51]. The same group also recently also demonstrated an iDEP-based high throughput and noninvasive strategy to directly distinguish Escherichia coli with compositional variations of lipopolysaccharides [51]. 

DEP manipulation has also progressed to much smaller biological particles such as virions and even molecules like oligonucleotides and proteins. The small dimension of protein and oligonucleotides makes them unfavorable for dielectrophoretic manipulation and the method is often seen as unsuitable due to the high applied voltages, required to compensate for the small particle radius. However, pioneering work by Washizu et al. [52] showed that efficient interaction is possible and meanwhile dielectrophoretic studies of a variety of oligonucleotides and over 20 different globular proteins have been reported. See the review in Ref. [53] and the publications cited therein. Furthermore, recent improvements in the fabrication of microelectronic systems [54] allow innovative electrode designs with optimized geometries and integration in microfluidic set-ups that enables the generation of strong enough ∇E^2.^.

An interesting aspect of DEP of globular proteins is that dielectrophoretic manipulation occurs at ∇E^2.^ several orders of magnitude lower than theoretically predicted [53].

Applying Equation (1) with the Clasius–Mossoti factor described by the bulk dielectric properties of the solute fails to explain dielectrophoretic manipulation of globular proteins. For example, the calculated value of ∇E^2^ required to generate a DEP force on Bovine Serum Albumin A (BSA) exceeding the random Brownian motions is in the order of 10^−21^ V^2^/m^3^. However, as illustrated in Table 1, DEP of BSA has often been carried out at much lower estimated ∇E^2^. We may therefore not consider the theory behind protein DEP to be complete and the mechanism behind it as well as a solid theoretical model remain still to be developed.

Recent work by Pethig and Hölzel [53,64] as well as Matyushov and Heyden [65,66] may give a more comprehensive explanation of protein DEP, however. Matyushov [65] suggested two reasons that contribute to the disagreement between theory and experimental observations: (i) a failure of Maxwell’s electrostatics to describe the cavity-field susceptibility, and (ii) the neglect of the protein permanent dipole by the Clausius–Mossotti equation. The magnitude of the dipole moment is given by the resultant of the moments of the distinct amino acids in the polypeptide chain, the moments of the charged acidic and basic groups about the molecules hydrodynamic center, and polarizations of the surrounding water molecules. A new theory was developed that included the cross correlation of the protein’s permanent dipole moment with its polarized hydration shell [66] and replaced the macroscopic CM factor.

**Figure 2:**
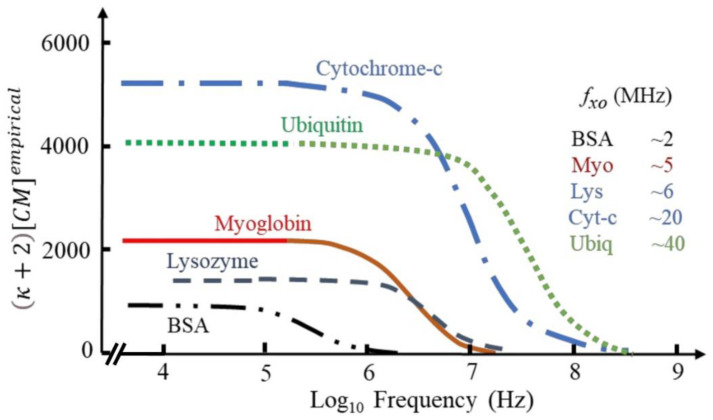
The frequency-dependence of the empirical factor (κ + 2) [CM] for a variety of proteins. Adapted under terms of the CC-BY license ref. [64] 2022, R. Pethig, published by MDPI.

In a series of papers, Hölzel and Pethig [53,64,67] propose a DEP force equation based on an empirical relationship between the macroscopic and microscopic forms of the Clausius–Mossotti factor. Like Matyushov, they also identified the intrinsic dipole moment of proteins as particularly relevant for DEP of globular proteins. The dipole moments of protein molecules, free to rotate about its prolate major and minor axes, manifests itself as a large dielectric dispersion (known as the β-dispersion). This dispersion was linked to the DEP effect, and they investigated if it could be used to predict the DEP interaction of proteins. Accordingly, they proposed that F_DEP_ can be predicted by using a correction factor (κ + 2) [CM] derived from the magnitude and frequency profile of its dielectric β-dispersion, which reflects the protein’s squared dipole moment and its relaxation time; whose estimation requires only a dielectric measurement over a limited frequency range. Subsequent MD simulation by Heyden and Matyushov supported this as they showed that the β-dispersion also encompasses cross-correlations of the protein dipole with its hydration shell [66]. The theory by Heyden and Matyushov or the empirical theory by Hölzel and Pethig can both be applied to predict the DEP response of a variety of proteins (Figure 2). Support for the accuracy of this predictions was further experimentally given by Liu et al. using the three model proteins immunoglobulin, α-chymotrypsinogen A and lysozyme [68]. The different proteins had their own DEP profile and were all shown to generate forces much larger than predicted by previous established theories but are consistent with the new theoretical framework. 

This new insight in the DEP of globular proteins that allows better estimation of the CM factor and the required ∇E^2^ will likely be very important for future work. It may allow a better understanding of DEP of small bioparticles as well as gain us more knowledge in how dielectrophoresis can be implemented in biosensors and protein detection.

## 3. Current Trends in Biosensors Assisted by Dielectrophoresis

### 3.1. Biomolecular Sub µm Sized Analytes

**Figure 3:**
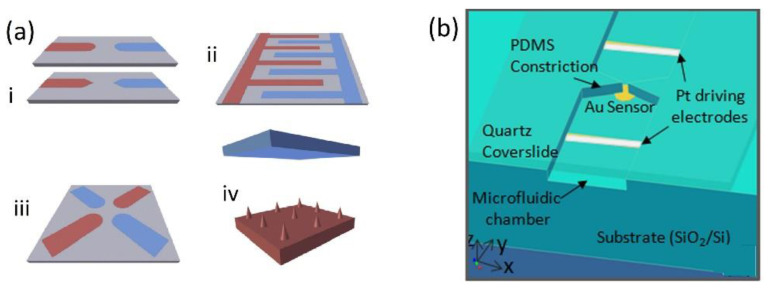
(**a**) Common electrode configuration for molecular eDEP (i) Electrode pair, (ii) IDE, (iii) quadrupole geometry, (iv) 3D geometry. Reproduced with permission [38] 2018, Elsevier. (**b**) Device geometry for the insulator constriction coupled to an electrically floating Au electrode sensor with immobilized DNA capture probe molecules. Reproduced with permission [70] 2012, AIP.

The implication of AC electrokinetic enrichment in biosensing has been demonstrated by several research groups using a variety of strategies and biomolecules such as oligonucleotides, antibodies, and globular proteins. For a more comprehensive list of examples, we refer to previous work [35] as we below only discuss some selected interesting examples. The limitations with almost all of these experiments, however, is that they have been performed in ideal solutions with low conductivity medium which highly restrains possible applications that has to be diluted before use. While the sensing abilities have been shown to be improved several orders of magnitude, most research still has to be considered as proof of principle experiments. To realize an eDEP effect in microfluidic set-ups there are four common electrode configurations in use (Figure 3a) [38]. The simplest configuration is a pair of planar electrodes facing each other, often with sharp edges to maximize the generated electric field gradients. Interdigitated electrodes (IDE) are often applied for efficient AC trapping as they allow the number of adjacent edges to be multiplied, enabling the covering of a large area while keeping a high field gradient. A quadrupole electrode arrangement based on two pairs of electrodes is generally applied for electrorotation experiments. Finally, also a 3D geometry in which the electrodes are positioned in different planes has been frequently applied. In this geometry, the electric field is characterized by being mostly built up perpendicular to the substrate, thus favoring DEP over AC electroosmosis [69].

DEP trapping using individual electrode pairs has been applied to improve nanowire and nanotube-based sensors multiple times. In a pioneering work, Gong et al. [71] reported a highly sensing performance of a nanowire FET with an electrode pair perpendicular to the nanowire, thus achieving AC-induced enrichment of the analyte at the sensor surface.

The applied AC voltage (0.5 V, 47 Hz) increased the sensitivity and the detection limit of PSA in water by a factor of 10^4^, thus allowing detection at concentrations in the attomolar range. The mechanism of this amplification was explained as a combination of AC electro-osmosis and streaming DEP.

**Figure 4:**
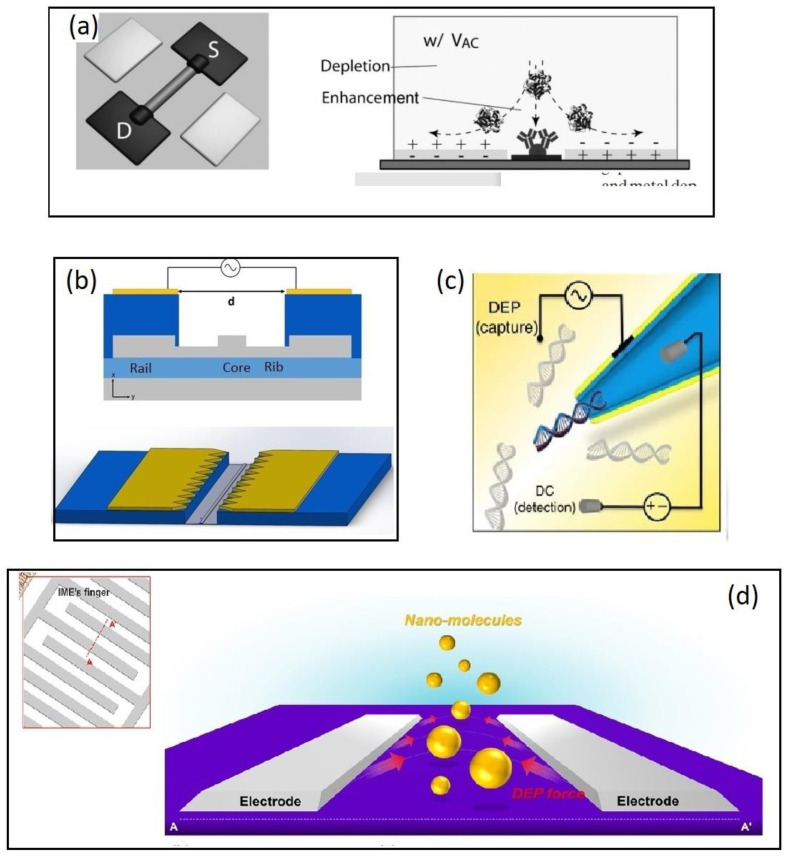
Examples of molecular biosensors combined with DEP for analyte enrichment. (**a**) A combination of DEP and electroosmosis enables a DEP interaction with proteins that cannot be immobilized against the electro-osmotic conveyance. Reproduced with permission [71] 2010, Wiley. (**b**) A SOI based microring resonator. The silicon waveguide (grey) is fabricated on the device layer of an SOI wafer while a coplanar electrode pair is placed on the metal 1 layer 1 um above the waveguide. Reproduced under terms of the CC-BY license Ref. [12] 2020, Henriksson et al. published by MDPI (**c**) Capillary nanopore sensor (CNP). An AC field between the metalized capillary and a planar electrode placed 20 µm away from the CNP. A separate DC field was simultaneously applied to allow the detection of DNA molecules. Reproduced under terms of the CC-BY license Ref. [79] 2016, Freedman et al. published by Nature publishing group. (**d**) A negative DEP force applied on an IDE pushes the analyte to the space between the electrode, which is functionalized with receptor molecules. Analyte capturing was monitored by impedimetric measurements. Reproduced with permission [80] 2016, Elsevier.

Streaming DEP refers to the focusing of particles into streams by equilibrating the DEP and drag forces acting on them [72] while electroosmosis is described as the interaction of the tangential component of the electric field and the induced charge in the diffuse double-layer on the electrodes [73]. Particularly when applying low-frequency AC voltages to a pair of co-planar electrodes in contact with an electrolyte, a fluid motion caused by the interaction of the electric field with its self-induced charges in the electrical double layer is generated [74]. The electroosmotic flow enables a microfluidic mixing that improves the transport of the analyte from the bulk solution (Figure 4a). The combination of the two forces, electro-osmosis and streaming DEP, enables a DEP interaction with proteins that cannot be immobilized against the electroosmotic conveyance, but are concentrated at the sensor surface [75]. While not the main contributing force, the authors also attributed some of the improved sensing to a favorable AC-induced intermolecular electrostatic interaction that enhances protein association after the electrokinetic application.

Similarly Sharma et al. [76] reported a single-walled carbon nanotube (SWCNT) immunosensor with two concentration electrodes facing towards each other and oriented perpendicular to the sensing electrode. The study focused on the regulatory protein of the myocardial contractile apparatus, Cardiac troponin, which was detected in sub picomolar concentrations. Dielectrophoretic concentration further lowered the detection time from 60 min to 1 min allowing the platform to be used for fast and sensitive diagnosis. The analysis was carried out in Tris/Borate/EDTA TBE medium (0.1 mS/m) and spiked human-serum samples at 5 V and 100–200 kHz frequencies. While a small deviation attributed to nonspecific bindings could be observed, the sensor platform showed high specificity over the interfering biomarker myoglobin, the TBE medium, and human serum and holds potential for use as a platform in biomedical diagnosis.

Li et al. [77] reported a capacitive immunosensor whose performance was substantially increased after focusing the analyte onto the sensor surface. The sensor simultaneously served both as a DEP electrode and a sensing electrode allowing detection of biomarkers for Johne’s disease in 10 ng/mL in about 2 min. The measurements were carried out using diluted PBS buffer (1 mM, 15 mM NaCl). 

The approach is also promising for the improvement of silicon photonic biosensors, as was recently shown by us. We designed and fabricated a Si-photonic biosensor based on the principle of evanescent detection of a ring resonator whose response is enhanced by focusing the analytes onto the active waveguide by DEP [12]. The ring resonator technology is based on the looped propagation of light in the form of whispering gallery modes, creating a resonance at frequencies fulfilling the resonance conditions. The waveguides are built on the device layer of a Silicon-on-insulator (SOI) wafer allowing electrodes to be prepared on either the device layer or at the back-end-of-line (BEOL). In the mentioned work, two different electrode configurations were designed. A coplanar electrode configuration in which the concentration electrodes are placed in the BEOL on the metal 1 layer on the chip facing each other at a 5 µm distance with the waveguide propagating in the gap (Figure 4b). Molecular analytes may then be focused onto the sensor following a mechanism similar to Gong et al., and larger analytes such as bacteria or algae may be focused directly onto the sensor surface by DEP. This was later shown to be successful in a similar approach by Petrowszki et al. [78].

In the second electrode configuration a 3D geometry bottom-up configuration was chosen so that highly doped silicon on the device layer makes one of the electrodes whereas the counter electrode is placed on top of a microfluidic channel. The advantage with this configuration is (i) the effect of electro osmosis may be reduced [69], (ii) the generation of a highly inhomogeneous electric field allows an electrode distance of 100 um, still generating ∇E^2^ values above 10^17^ V^2^/m^3^ according to simulations [69], and (iii) analytes may be focused within a micrometer from the active part of the sensor surface.

A 3D electrode geometry was also applied by Freedman et al. [79] (Figure 4c) as they recently reported a 1000-fold improved detection efficiency in nanopore sensing by incorporating a DEP trap at the nanopore opening. Conductive nanopipettes (CNP) or nanopores are capillaries of glass or quartz with diameters down to sub-10 nm whose inner wall is coated with a layer of a conductive material and designed so that a nanometer-sized aperture is formed at the tip. The exclusion of ions when the biomolecule enters the capillary causes a decrease in bulk ion, allowing single molecules to be detected [81].

Freedman et al. realized an efficient DEP trap for concentrating the analyte by applying an AC field (10–20 V and 0.5–4 MHz) between the metalized capillary and a planar electrode placed 20 µm away from the CNP. A separate DC field was simultaneously applied to allow the detection of DNA molecules (double-stranded 10 kbp,) at concentration as low as 5 fM. The limitation with the experiments is again the non-physiological medium applied (water, conductivity not available) as well as the large DNA molecules. However, under these conditions the set-up seems remarkable sensitive.

Negative DEP applied to improve the sensing performance of interdigitated electrode IDE-based impedimetric biosensor platforms has been explored in a series of publications by Hwang and co-workers [80,82,83,84]. The principle behind the negative DEP (nDEP) enrichment of analytes is that analytes are repelled away from the electrode in the direction of the active site of the sensor surface where they are trapped between two electrodes fingers (Figure 4d). Hwang and coworkers used an IDE electrode comprised of 30 pairs of microelectrode fingers (spacing, 5 µm length, 300 µm) and an impedimetric detection strategy in which an AC field of 10 mV generates the highest electric field gradients on the edges of the electrodes [80]. As analytes approach the electrode edges, they are directed towards the space between the electrodes via nDEP and subsequently captured by immobilized receptor molecules. The applied AC voltage of the IDE electrode both serves to generate the DEP force as well as transducing the bio interaction event. The group has focused on the detection amyloid-beta and Tau as biomarkers for early-stage Alzheimer diagnosis and showed results with successful detection of biomarkers in concentration as low as 100 fg/m [83].

The AC enrichment resulting in two-fold increase in sensitivity seems to be less efficient compared to the previous studies applying positive DEP, however. A likely explanation is the relatively small voltage applied that only locally generate strong enough electric field gradients. The electric fields do not extend far from the sensor to the solution and the analytes must in principle diffuse to the electrode surface in order to interact with the electric fields. The authors concluded that higher voltages would push the analytes away from the sensor surface and a tradeoff and optimization of the applied voltage is necessary to achieve the highest possible enrichment. On the other hand, this could also be used as an advantage as larger matrix particles could be pushed away from the sensor surface due to stronger DEP interaction while concentrating the amyloid beta molecules [83]. While the two-fold improvement in sensitivity of amyloid-beta may seem relatively small, it must be considered very useful at these low levels of concentration. It is, however, unclear in how far the approach may be transferred to detect other analytes, as there are only a few examples of proteins exhibiting a cross-over frequency, and negative DEP so far only have been presented on BSA, avidin, prostate-specific antigen, and amyloid beta. Nevertheless, the high sensitivity of the sensing platform makes it a valuable tool, and one way to make it available for more analytes could be to use functionalized poly beads. Such approach was also successfully shown by the same authors [82].

In addition to the work above, nDEP enrichment of biomolecules has been achieved by iDEP approaches [85,86,87]. An example is the work by Rohani et al. [85] as they used the device to focus biomolecules into a nanoslit structure, for ultrafast sensing. They applied a DC-offset AC field given a strong ∇E^2^ realized by lateral constrictions fabricated inside the nanoslit structure. Enrichment of biomolecules occurs under a force balance of nDEP versus electrophoresis in the trapping region. Accordingly, selectivity is achieved both due to the polarizability and interaction with the ∇E^2^ as well as due to surface charge affecting the balancing force due to electrophoresis. The researchers presented with this set-up a frequency-selective enrichment of PSA versus anti-mouse IgG antibodies in the 4–6 MHz range. By coupling enrichment to voltametric detection using immobilized receptors on graphene-modified surfaces, they showed that the false positives usually obtained with >10^4^-fold higher levels of interfering anti-mouse IgG antibodies can be almost eliminated. A particular great advantage with this approach is that the set-up was used in solutions with conductivity corresponding to physiological media, as the electrodes in an iDEP approach are not directly exposed to the solution electrochemical side effects and joule heating can be reduced. This makes the approach probably most feasible, in order to take the step from proof of principle experiments to sensing real samples. 

### 3.2. Detecting Microbial Analytes 

The effect of diffusion and mass transfer limitations in receptor-based biosensors become particularly significant when attempting to register larger bioparticles such as whole cells and bacteria, since few analytes are present in the sample solution (<10^5^ colony forming units (CFU)/mL in clinically relevant samples) and these bind less efficiently to the sensor surface. Furthermore, the larger size of the analyte affects the efficiency of diffusion/Brownian movement, and the analysis of cells may be further limited if the cells are actively moving. The target cells are generally delivered to the sensors via microfluidic solutions with microchannel heights of tens to hundreds of micrometers; consequently, most cells will pass the microchannel without adsorption on the sensor surface. As cells interact strongly with an applied AC field, sensing may be substantially improved by DEP, with less required voltage compared to molecular analytes. Accordingly, several works have been devoted to the topic for a variety of sensing platforms.

As excellently reviewed by Fernandez et al. [34], DEP has been frequently applied to enhance microbial detection for a variety of applications, often with a detection limit improved by several order of magnitude reaching levels as low as 10^2^ CFU/mL.

The coupling of impedimetric measurements with DEP using interdigitated electrode arrays has been shown to be particularly promising for detection of a variety of bacteria as well as enabling selectivity over non-viable cells and non-target strains [48]. The geometry of the interdigitated electrodes is very suitable to enable highly sensitive detection, as (1) the common electrode gaps are on the order of a microbe and (2) the geometry allows the generation of a strong DEP force.

Using pDEP enrichment, the bacteria are focused on the edges of interdigitated electrodes functionalized with antibodies, bacterial capture was found to be 3–5 fold higher than for immunocapture without DEP enrichment. A recent example is the work by Muhsin et al. [88] who focused bacteria onto an antibody functionalized IDE array, thus achieving selective detection of living legionella cells in water samples in concentration of 3 cells/mL. Similar result was also reported for capacity sensors based on single wall carbon nanotubes (SWCN) [89].

**Figure 5:**
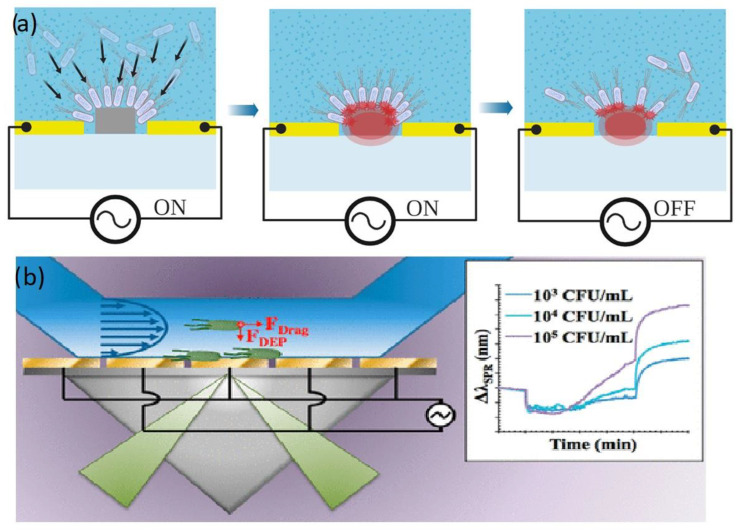
(**a**) Capture of E.coli on a silicon waveguide. Reproduced under terms of the CC-BY license Ref. [78]. (**b**) Schematic figure of a microbial SPR sensor, reproduced with permission [90] 2018, American Chemical Society.

Recently, DEP-enhanced microbial detection has further been expanded to photonic sensors. A promising example is the study by Galvan et al. [90] who substantially improved the sensitivity in detecting living bacteria with surface plasmon resonance (SPR). Dually functional IDEs capable of sustaining SPR and generating DEP into a single iSPR chip were applied to lower the LOD of E.coli in water samples by nearly five orders of magnitude (∼3.0 × 10^2^ CFU/mL) compared with conventional SPR chip without DEP that reached an LOD of only ∼1 × 10^7^ CFU/mL (Figure 5a). 

Another recent example of a photonic biosensor with DEP analyte trapping is the work by Petrowszki et al. [78] who were able to detect E.coli at similar concentration on a silicon waveguide sensor. The research team fabricated gold electrode pairs on the rail of a rib waveguide on the device layer of the SOI, enabling a narrow bandgap and a strong electric field that focused the E.coli onto the core waveguide. Their device with assisted DEP was shown to outcompete their previously silicon photonic sensors [91]. (Figure 5b).

### 3.3. Avoiding Nonspecific Bindings in Biosensing with Dielectrophoresis

Biosensing approaches generally rely on some form of receptor molecule that specifically and selectively recognizes a target analyte whose binding is transduced to an electric signal that is further processed and yields information about the bioparticles captured and their concentration. Thus, it is crucial that the transduced signal correspond to the analyte of interest and is not derived from nonspecific binding of interfering particles. 

The specific binding generally occurs at specific binding sites and is characterized by (1) short-range forces such as hydrogen bonds and van der Waals/acid−base interactions, (2) a defined final interface and orientation, (3) evolved or designed binding sites, (4) high affinity [92].

Nonspecific bindings generally have a much lower affinity to the receptor, and the binding is characterized by long range forces like electrostatic and hydrophobic with poorly defined surface orientation. The binding may be reversible or permanent, involving a structural conformation change. While the target analyte generally exhibits a much higher affinity to the receptor than other interfering particles, if the concentration of the latter is magnitudes higher than the analyte, the sensor surface can still be dominated by nonspecific binding of undesired particles, suppressing the signal from the analytes. Furthermore, many receptors such as antibodies are often susceptible to cross-reactivity, allowing a number of molecules to bind to the same binding site on a receptor surface even though they should ideally be specific for a single target [92].

One way to avoid nonspecific binding is a suitable sample preparation strategy that removes interfering particles. Removal of interfering compounds or microbes by pretreating the sample can increase the sensitivity of the system by increasing the ratio of the interesting low abundance analytes to the interfering bioparticles of much higher abundances. Another potential strategy would make use of DEP that can be a suitable tool for sample preparation, especially for sorting out target microbes or cells from complex matrix such as blood plasma, urine, or saliva. One successful example has been the implication to increase the sensitivity of PCR blood analysis by separating the few target bacteria from interfering blood cells, as reviewed in detail by Fernandez et al. [34].

The pathogen concentration in a sample of an infected patient can be just 1–100 CFU/mL while a µL blood contains 4–6 billion blood cells, whose components such as heparin and hemoglobin inhibit PCR amplification. Blood cells were separated from the target pathogen, often with IDE that induce an nDEP effect on the blood cell and a weak pDEP on the pathogens at low frequencies and thus enable a separation. The approach was successfully demonstrated for PCR and a variety of samples containing various bacteria. For instance, Cai et al. applied it for pretreatments of samples with 20 different pathogens [93].

Similar approaches can be easily transferred to biosensing platforms, for example to separate soil particles from bacteria in environmental samples [94], or to separate viable and nonviable cells before the detection [95]. An increase in the conductivity of dead cell membranes by factor 4 is responsible for this separation [96].

While more complex and harder to predict, streaming DEP can also be applied for preparation of biomolecular samples such as DNA isolation prior to PCR [97] or to reduce nonspecific bindings in immunosensors. A recent example is the work by Kim et al. as discussed in Section 3.1, who applied DEP-based filtration for the detection of amyloid beta in diluted plasma samples for diagnosing Alzheimer’s disease [83]. Their device used an IDE electrode with 5 µm spacing and an AC field of 10 mV and 50 Hz based on the relative strength between the difference in DEP forces applied to target molecules and matrix factors caused a filtration effect that reduced nonspecific bindings of the subsequent IDE-based immunoassay with up to 50%.

**Figure 6:**
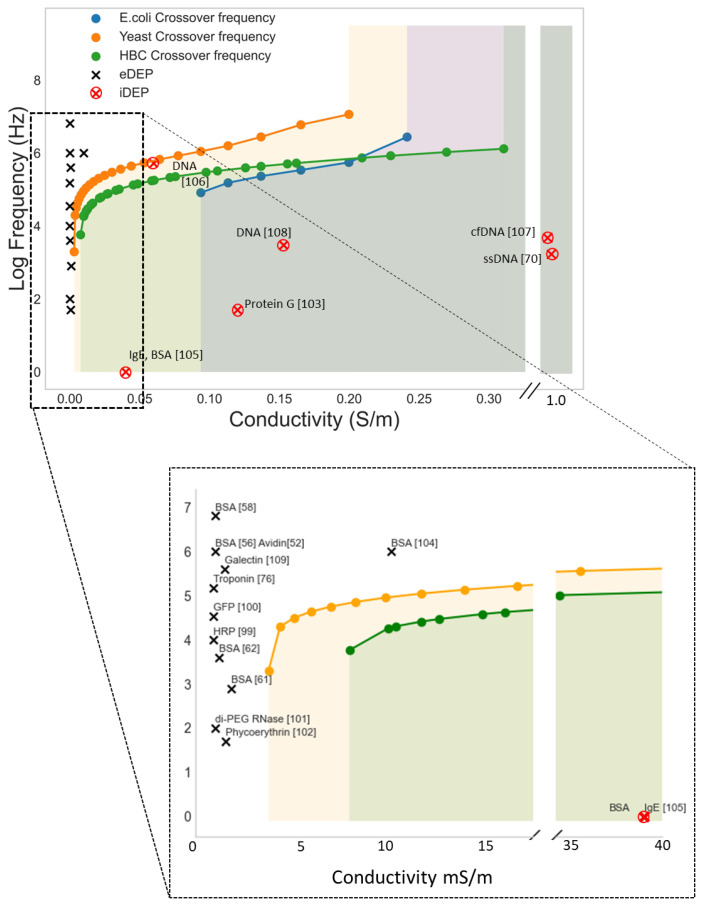
Simulated lower cross-over frequencies (nDEP to pDEP) for E.coli, Yeast cells (Saccharomyces cerevisiae), and Human B cell (HBC) as a function of medium conductivity σ_m_. The cells’ crossover frequencies were calculated with the software MyDEP [98]. The black and red marks shows pDEP conditions reported in the literature for a variety of biomolecules [52,56,58,61,62,70,76,99,100,101,102,103,104,105,106,107,108,109]. The colored areas display conditions below the cross-over frequency (medium conductivity and frequencies) where the cells experience nDEP. Accordingly, pDEP of biomolecules in this area would enable an efficient separation.

An ideal approach would be DEP conditions where the biomolecules are focused onto the sensor surface while larger interfering cells are pushed away. Figure 6 shows the crossover frequency from nDEP to pDEP for E.coli, yeast cells, and Human B lymphocytes as well as examples of pDEP conditions for various biomolecules. The colored areas represent conditions (medium conductivity and frequencies) where the microbes experience nDEP and accordingly pDEP of biomolecules in this area would enable an efficient separation.

As shown in the Figure 6, however, most work on molecular DEP has been carried out at low frequencies and in media of low conductivity, precluding such an approach. Molecular DEP can be carried out in a frequency range between 1 kHz to 10 MHz but are generally carried out at low frequencies as it lower the ∇E^2^ threshold required for DEP interaction [52]. Low ion strengths medium is used as increasing the conductivity of the medium over 1 mS/m will generally cause excess joule heating and electrochemical reactions on the water-electrode interface [110]. At this low medium conductivity, biomolecules as well as larger cells, microbes or particles will experience pDEP below 10 MHz. As a consequence, for pDEP enrichment of biomolecules the samples need to be diluted and moreover completely purified from larger particles, as they will interact much more strongly with the applied AC field, blocking the receptor molecules.

**Figure 7:**
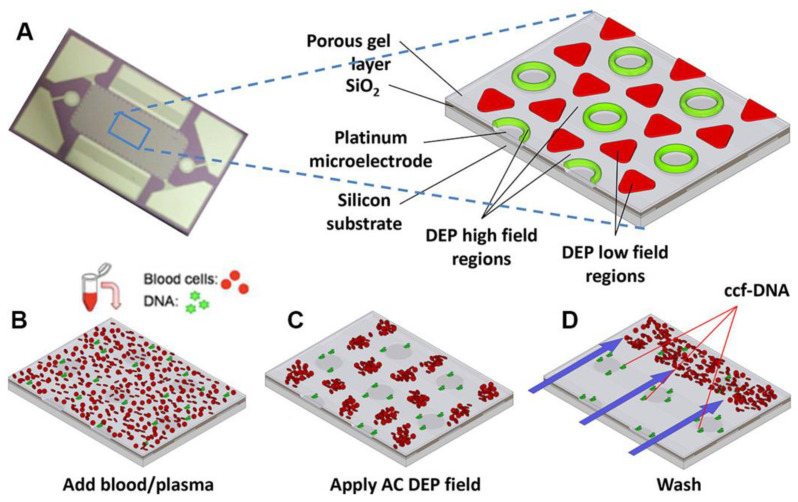
DEP microarray device and scheme for isolation of ccf-DNA from blood and plasma. (**A**) Upper image shows the alternating current electrokinetic microarray device (chip) used to carry out the isolation of ccf-DNA directly from blood. Expanded view shows the device materials composition: porous gel, platinum microelectrodes, SiO_2_ layer, and silicon base; and the location of the DEP high-field (green) and the DEP low-field (red) regions when an AC field is applied. Lower figure shows (**B**) microarray with whole blood (red circles) containing fluorescent DNA (green dots); (**C**) application of the AC electric field causing the fluorescent DNA (green dots) to be concentrated in the DEP high-field regions on the microelectrodes, while the blood cells (red circles) move into the DEP low-field regions between the microelectrodes; and (**D**) the AC field remains on while a fluidic wash removes the blood cells from the microarray with DNA remaining concentrated in the DEP high-field regions. Reproduced with permission [107] 2014, Wiley.

One potential way around this obstacle may be to apply an insulator DEP (iDEP) approach. With this approach it is possible to bypass the requirement of a low conducting medium and there are several examples of molecular pDEP of both oligonucleotide [70,107,110,111] and proteins [105] in moderate conducting medium. The main advantage with iDEP is fewer problems with electrochemical side effects. The work by Sonnenberg et al. [107] is particularly interesting for biosensing application as they realized an array of microelectrodes that were able to focus heigh weight DNA (20–200 kbp) on the high field areas of the chip with a pDEP force while a nDEP force moved blood cells to the low field areas (Figure 7). If the set-up can be adapted to also immobilize analytically more relevant lower weight biomolecules, it will be a great step towards higher sensitivities.

A second key factor to avoid nonspecific binding in affinity sensors is a suitable biofunctionalization strategy that allows for reproducible immobilization of receptor molecules. This is often realized in two steps involving an interlayer between the receptor molecule and the surface formed by a self-assembling process [112]. 

DEP has also been applied for this purpose to immobilize viruses [113] or biomolecules [69,100] with retained functionality and could be an alternative to conventional biofunctionalization strategies for certain applications. For instance, Otto et al. [69] immobilized IgG irreversibly onto a CMOS compatible silicon-based chip device with a regular array of more than 10^6^ cylindrical sub-microelectrodes. They showed that the antibodies were selectively immobilized onto the electrodes and that they retained their functionality. An advantage with the method is that you can selectively functionalize only the desired functional area on the chip, thus avoiding receptor being immobilized outside the transducer whose analyte interaction cannot be registered. Such specific biofunctionalizations of heterogeneous surfaces are otherwise fairly complicated but important to push down sensitivity and LOD.

For instance, silicon-based sensors are often fabricated from a silicon-on-insulator SOI wafer where the sensing elements and possible integrated circuit devices are built on the device layer, with the buried oxide enclosing the device, functioning as an effective etch-stop during wafer processing. Therefore, by applying state-of-the art silane-based biofunctionalization approaches that react with the native oxide layer on the Si surface, the receptor molecules will be immobilized all over the chip and not specifically at the sensing region. Biointeraction events that take place at the enclosed oxide region will not be detected, thus potentially reducing the sensitivity and LOD. Work devoted to the functionalization of SOI devices focusing on selective functionalization of the silicon surface over SiO_2_ could therefore improve the performance of the sensors. The methods available today such as electrografting of diazonium salts [114], hydrosilylation [115,116], and arylation [117] reactions still require optimization [118] and a hydrogen terminated surface and inert atmosphere. DEP-induced immobilization of receptor molecules offers an additional simpler protocol to achieve selective biofunctionalization of heterogenous surfaces. 

It is also crucial that the interface orientation of the receptor molecules is available for interaction with solutes. If the binding site is inaccessible to the analytes for example due to steric hindrance and low-quality interlayers, specific interaction with the analyte is precluded and nonspecific bindings may be facilitated. For instance, while immobilizing antibodies it is crucial that the molecule is oriented with the paratope pointing upwards and the end of the FC region is bonded to the surface. For site-specific immobilization on surfaces, various immobilization methods have been developed. This included strategies based on Histidine or FLAG tags [119] and click chemistry [120]. The tagged biomolecules are then conjugated to an interlayer on the surface via a coupling reaction. 

**Figure 8:**
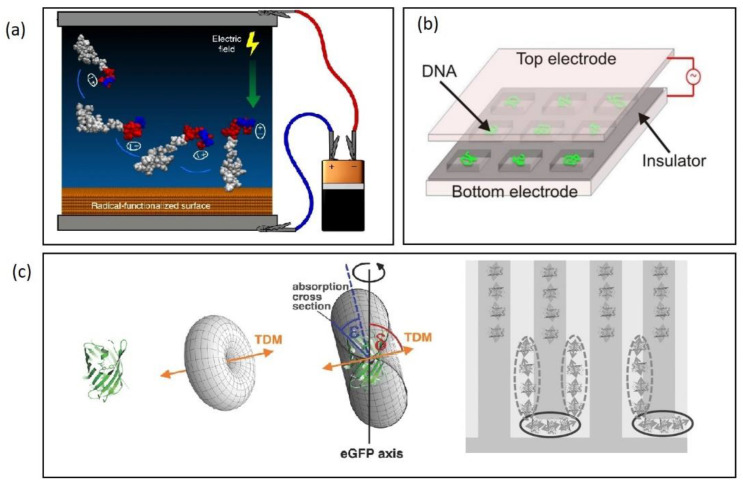
(**a**) Electric fields to control the orientation of peptides bonded to surfaces. Reproduced under terms of the CC-BY license Ref. [122] 2018, Martin et al., published by Nature publishing. (**b**) AC stretching of DNA in top-bottom electrode set-up. Reproduced under terms of the CC-BY license Ref. [126] 2018, Martin et al., published by Nature publishing. (**c**) Aligned immobilization of GFP onto an IDE electrode. Reproduced with permission [100] 2016, Wiley.

Additionally, electric fields can assist to achieve a controlled surface orientation of immobilized biomolecules as well as an improvement in the quality of the structure ordering of the self-assembled probing molecular interlayer. For example, the structural ordering of APTES monolayers covalently bound on a silicon nanowire surface was shown to align to the direction of an applied electrical field of 0.5 V [121]. This field-alignment technique was shown to improve the binding efficiency of Ag nanoparticles and the biofunctionalization of 15-mer ssDNA molecules enhancing sensitivities by three orders of magnitude. Control of the orientation of surface-bound peptides has been achieved by tuning the electric fields at the surface during immobilization, thus inducing a spontaneous irreversible immobilization when the peptide made contact with the surface [122]. Both peptide orientation and surface concentration could be controlled simply by varying the solution pH or by applying an external electric potential of 5 V delivered by a small battery (Figure 8a). Furthermore, the use of DC field up to 8 V was shown to control the orientation of antibodies, which resulted in more than 100% enhancement in signal-to-noise ratio compared with normal physical adsorption [123]. Likewise, AC fields have been applied to control the surface orientation of immobilized biomolecules via DEP-induced immobilization. Laux et al. immobilized green fluorescent protein, GFP onto an IDE by DEP by applying an AC voltage (13 V_rms_ at 100 kHz) and showed that the alignment of immobilized protein follows the molecules’ geometrical shape with their longitudinal axes parallel to the electric field, allowing a prediction of the surface orientation of the molecules (Figure 8c) [100].

An open question is how the immobilized biomolecules are affected by the applied AC field. Whether the receptor is immobilized via DEP-induced physical adsorption or via a coupling reaction to an interlayer monolayer, they will interact with an applied electric field. An applied AC field to realize analyte enrichment may potentially influence the receptor conformation and thus either increasing or decreasing its reactivity. DC fields have previously been applied to control reactivity of receptor molecules. For example, it was shown that it is possible to affect the conformation of IgGs by pushing or pulling electrostatically Fab fragments towards or from the electrode surface [124]. Here, a potential difference between electrode and solution acts on IgGs’ charged amino acids and enable tuning the accessibility of the paratope. They showed that antibody–antigen affinity is affected by the sign of the applied potential. Thus, a positive potential enables an effective capture of antigens, whereas a negative potential pulls the fragments towards the electrode. The steric hindrance thus largely hampers the antigens capture. Similarly, a molecular surface was designed and developed that utilizes an electric potential to drive a conformational change in surface bound peptide moiety, to give on-demand control over antigen−antibody interactions on sensor chips [125].

Dielectrophoretic forces have similarly been shown to be suitable to stretch DNA and other nucleotides for example between interdigitated electrodes [127], in top bottom geometry [126] or using electrode pairs [128]. Being able to control the stretching of DNA in this way may allow significantly improved hybridization kinetics and hybridization efficiency of DNA based biosensors due to faster diffusion and less steric hindrance [129].

Important to consider is the possible irreversible negative effect an electric field may have on the biomolecular layer and its functionality. Li et al. [130] studied the effect of an applied electric field on self-assembled monolayer of organic molecules on oxide-free silicon surfaces. They reported that typical monolayers on hydrogen-terminated silicon undergo partial desorption followed by the oxidation of the underneath silicon at +1.5 V vs Ag/AgCl. Furthermore, the monolayer lost 28% of its surface coverage and 55% of its electron transfer rate after 10 min. Such a reduction in surface-bonded receptor molecules will highly affect both the analyte capture ability as well as promote nonspecific binding. Accordingly, biofunctionalizations strategies need to be compatible with the applied AC field.

The biological function of a protein is highly dependent on its three dimensional structure and the latest research clearly shows that an electric field sometimes as low as 500 V/m can promote disturbances on protein conformation, change their unfolding mechanisms, aggregation, and interaction patterns [131]. As a matter of fact, electric field technologies are seen as serious alternatives to traditional thermal processing [131].

For example, Bekard et al. [132] studied the conformation of bovine serum albumin and lysozyme at an AC field with frequencies between 10 and 500 Hz and field strength of 0.78 to 500 V/m at exposure times up to 3 h. They concluded that the electrophoretic motion associated with the alternating field breaks the hydrogen bonds in the protein tertiary structure, thus the protein is unfolded. Especially at low electric field strengths, the applied frequency seems to be an important factor for protein unfolding, in which unfolding is more pronounced at lower frequencies [131].

Another example is the SARS-CoV-2 spike protein and its affinity to the ACE2 receptor. The binding of the spike protein with the ACE2 receptor (ACE2) of the host cell constitutes the first and key step for virus entry. Molecular dynamic simulations shows that electric field of about 10^5^ V m^−1^ which is commonly applied in protein DEP, may result in substantial conformation changes, thus likely reducing the binding affinity [133]. Based on previous work showing that mice exposed to an electric field of about 10^4^ V/m to two weeks experienced no negative effects [134], the author suggested that electric fields at this magnitude may be suitable for in vivo or in vitro therapeutic approaches. While this could be a possible route for treatment, it would preclude biosensor platforms with immobilized ACE2 receptors [135] being exposed to an electric field.

## 4. Perspectives and Challenges

While DEP-assisted biosensing has caught some attention in the last decade, the approach is still surprisingly unexplored given the potential of the technology that may improve the sensitivity by several orders of magnitudes. The approach is still immature with several challenges to be addressed but also offers many opportunities as listed in Table 2. 

One obvious challenge when coupling biosensors with DEP is the requirement of a more advanced system integration. Additional electrodes need to be integrated in the flow cells increasing the costs and time to be invested in the fabrication of the sensor and microfluidic system. Another major disadvantage with DEP as a biosensor enhancer is the requirement of a low conductive medium for efficient pDEP. In most of the published work, pDEP is realized by a variety of electrodes solutions, based on electrode pairs, IDE, or 3D set-ups to realize a DEP-induced analyte enrichment at the sensor surface. 

Natural biological conducting media generally precludes positive DEP for most bioparticles. Therefore, to detect cells in standard non-diluted buffer solutions a system based on nDEP induced enrichment, is likely inevitable for most analytes. The nDEP induced analyte enrichment strategy studied by Kim et al. [80] would be an example of such approach, the drawback here is that there is a trade off in the applied voltage, as too high voltages will push the particle away from the sensor.

We expect DEP-assisted biosensors to be most beneficial for microbial analysis in low conducting medium, such as detection of legionella bacteria in drinking water. For instance, since 2011 there is a legal obligation in Germany to examine for legionella in all drinking water installations with a central flow heater and in hospitals, homes for the elderly, and sports facilities, resulting in a correspondingly high level of commercial interest. So far, the Legionella detection has been carried out with standard culture methods, in which a final result takes ten days. Using molecular biological methods, such as PCR, the analysis times can be shortened considerably, but they cannot distinguish whether the detected biomolecules are from living (and thus reproductive) organisms or from dead cells. The technique also requires equipped diagnostic laboratories, trained personnel, and cost-intensive consumables.

The DEP technology on the other hand is characterized by simple handling and the possible differentiation between living and dead cells [136] enabling only clinically relevant cells to be separated from the sample and subsequently quantitatively detected. The understanding of DEP on microbials is comprehensive and the route to commercialized products appears straight forward.

As shown in this review, the sensing of protein or nucleic acids can also benefit from DEP enrichment. Sensitivities and response time can be improved by orders of magnitude, and several works on single molecules highlight its potential. The limitation with most of the work is that they have been carried out in non-physiological model solutions without interfering solutes, and regarding its implication as molecular biosensor, we consider it a high-potential approach that is still immature and rather distant from commercial prototypes. Since the pioneering work by Gong et al. [71], the number of published works that combines biomolecular sensing and DEP are relatively sparse. This indicates that it may be challenging to take the step from proof of principle research to more realistic experimental conditions.

As pointed out in recent reviews by Frutiger et al. [92] and Wilson et al. [20], nonspecific bindings is considered the most important factor determining the sensitivity and LOD in biosensors. This is especially true for immunosensors since their target specificity is lower than, for example, DNA-hybridization-based sensing. Accordingly, DEP enrichment should be specific for the desired analyte to achieve an increase in sensitivity or to lower the LOD. The DEP effect can be tuned by changing the amplitude or frequency. Nevertheless, the small sizes of biomolecules make DEP challenging, and while DEP separation of biomolecules have been reported it is still an open question in how far specific DEP interaction can be achieved in real samples. The total force on the proteins is given by the sum of many forces including sedimentation, Brownian, dielectrophoretic, and hydrodynamic forces as well as fluid flow induced by electroosmosis whose magnitudes can be of the same order as, or sometimes even larger than, the DEP force [73]. This makes predictions even harder. If a sample contains a large number of interfering particles that also are transported to the sensor surface due to the DEP force, the sensor surface will be covered with undesired particles, preventing an increased performance. To realize a pDEP effect in an eDEP set-up, the conductivity of the solution has previously not been higher than 10 mS/m. At this low medium conductivity, biomolecules as well as larger cells, microbes, or particles will all experience pDEP over a large frequency range. As a consequence, when applying pDEP enrichment of biomolecules the samples need to be diluted and moreover completely purified from larger particles, as the DEP force correlates with the cubic of the particle radius the interaction with larger particles such as cells will be great and trapped cells may accumulate and block the receptor molecules.

A pDEP force can be applied to permanently immobilize biomolecules, which could be beneficial specially to realize selective bonding, for example on the silicon-based device layer in SOI devices. However, such physical adsorption needs to be avoided during receptor analyte interaction to allow selective bonding. An enrichment of biomolecules by positive eDEP, therefore, seems to be best realized by an nDEP approach similar to the one suggested by Kim et al. Recent published work [68,83] that allows separation of proteins of different shapes and sizes as well as their conformity with new developed theory is encouraging, however. 

The new insights in the mechanism of protein DEP will be valuable in the endeavor for a better understanding as knowledge of the variables in the CM function will allow more accurate prediction of the DEP interaction for a variety of proteins. In this way, we may identify the most promising system for DEP-assisted molecular biosensing. When making these predictions, the ∇E^2^ generated by the electrodes is another parameter that is important, especially for biomolecules as higher fields optimized for the application are required to overcome the other forces acting on the biomolecule. While the ∇E^2^ can be directly derived by FEM simulation to obtain a rough picture, it will still deviate from real values, as fabrication tolerances on the electrode structures are generally not included. Furthermore, the importance of correct impedance matching and the voltage drop inside DEP actuators should be taken into account when predicting the DEP effect of the device [137,138].

The influence of the electric field on the activity of both receptor molecules as well as the analytes should be investigated more deeply. A high-quality and well-oriented layer of receptor molecules is important for high sensing performance as defects may hamper analyte receptor interactions and facilitate nonspecific binding. It was shown that typical monolayers on hydrogen-terminated silicon undergo partial desorption followed by the oxidation of the underneath silicon at +1.5 V vs. Ag/AgCl. Furthermore, the monolayer lost 28% of its surface coverage and 55% of its electron transfer rate after 10 min [130]. Detaching of receptor molecules as well as conformational changes of receptors or analytes could be important limitations. On the other hand, electric fields may also potentially improve the monolayer quality and the accessibility of the binding sites, for instance by applying an nDEP force acting on the receptor molecules. This was realized for DNA stretching, and similar approaches might be beneficial to increase the accessibility of immobilized antibodies or enzymes.

As pointed out, iDEP can be applied for enrichment of biomolecules via nDEP or pDEP in higher conductive medium that is more relevant for bioanalysis. Furthermore, pDEP of heavier biomolecules have been shown in solutions where many larger bioparticles such as blood cells and bacteria experience a nDEP force. This variation could be utilized for more targeted DEP to move desired proteins or nucleic acids to the sensor surface while repelling interfering cells in plasma samples or in a bioprocess environment. 

While not covered in this review the use of biofunctionalized pollybeads that can be dragged to the sensor surface by DEP have shown to be beneficial for improved biosensing. The µm-sized particles interact strongly with the AC field and the approach could be an alternative to the use of magnetic nanoparticles [82,139] and their dragging to the sensor surface by magnetic forces.

## 5. Conclusions

In this review, we have aimed to cover opportunities, perspectives, and challenges regarding the implementation of DEP to enhance the performance of biosensors.

We can see that the approach has obvious advantages in application aiming to detect whole cells such as bacteria or to separate target biomolecules from larger interfering particles such as blood cells. Detecting whole cells via an immunoassay requires actions to drag the analyte to the sensor surface, and pDEP seems to be a very suitable approach to achieve this. pDEP of microbes and cells is limited to low conducting medium, however, and the most promising applications seems to be analysis of drinking water samples.

Applying DEP to focus biomolecules onto the sensor surface, may potentially improve response time and LOD within reasonable time scales; however, the research is still in a state of fundamental research. While increased sensitivity of various devices has been reported as well as protein separation that can be useful to avoid non-specific bindings, the limitations of most of the work so far is the requirement of a low conducting medium, and taking the step further from proof of principle experiments will be challenging. iDEP approaches that use either pDEP or nDEP to focus the analytes may be the most feasible solution in this endeavor.

## Figures and Tables

**Table 1:** Published parameters for DEP manipulation of BSA. ∇E^2^ estimated by Hayes [55].

DEP Method	Estimated ∇E^2^ (V^2^/m^3^)	Frequency (kHz)	Medium Conductivity (mS/m)	Reference
eDEP	10^18^	10–30,000	<1	[52]
eDEP	10^19^	1000	0.2	[56]
eDEP	10^19^	100	3	[57]
eDEP	10^18^	2500	0.001	[58]
eDEP	10^21^	10	0.001	[59]
iDEP	10^12^	DC	2.5–10	[60]
iDEP	10^18^	DC	10	[61]
iDEP	10^18^	100	0.28	[62]
iDEP	10^23^	1	80–2000	[63]

**Table 2:** Opportunities and challenges of DEP assisted biosensing.

Biofunctionalization	Capture of Target Molecules	Receptor Analyte Interaction
+ Selective surface functionalization + Engineerable surface orientation of immobilized biomolecule. − Surface orientation of receptor determined by the orientation of its dipole moment − Physical adsorption, risk of loss of activity	+ Improved mass transfer of proteins and microbes. + Analyte enrichment may increase LOD with orders of magnitude. + Selective interaction with target particle is possible, at least for larger particles. + iDEP may allow a separation between target biomolecules and interfering larger particles such as blood cells. − Difficult to realize electrode-based pDEP in physiological medium. − Weak interaction of biomolecules with the AC field due to their small sizes.	+ Possible improved accessibility of receptor, for example by DNA stretching, improved monolayers − Possible desorption of receptor molecules − Possible inactivation of receptor or analyte. − Possible DEP induced nonspecific physical bonding of analytes.

## Data Availability

Not applicable.
